# Associations Between Social Determinants of Health and Adherence in Mobile-Based Ecological Momentary Assessment: Scoping Review

**DOI:** 10.2196/69831

**Published:** 2025-09-23

**Authors:** Yinan Sun, Aditi Jaiswal, Christopher Slade, Kristina T Phillips, Roberto M Benzo, Peter Washington

**Affiliations:** 1 Department of Information and Computer Sciences, University of Hawaiʻi at Mānoa, Honolulu, HI, United States; 2 Department of Health Systems Science, Kaiser Permanente, Honolulu, HI, United States; 3 Kaiser Permanente Bernard J Tyson School of Medicine, Pasadena, CA, United States; 4 Division of Cancer Prevention and Control, Department of Internal Medicine, College of Medicine, The Ohio State University, Columbus, OH, United States; 5 Department of Medicine, Division of Clinical Informatics and Digital Transformation (DoC-IT), University of California, San Francisco, 10 Koret Way, San Francisco, CA, 94143, United States, 1 (415) 353-2067

**Keywords:** ecological momentary assessment, social determinants of health, digital health equity, mHealth, scoping review, PRISMA

## Abstract

**Background:**

Ecological momentary assessment (EMA) involves repeated prompts to capture real-time self-reported health outcomes and behaviors via mobile devices. With the rise of mobile health (mHealth) technologies, EMA has been applied across diverse populations and health domains. However, the extent to which EMA engagement and data quality vary across social determinants of health (SDoH) remains underexplored. Emerging evidence suggests that EMA adherence and data completeness may be sometimes associated with participant characteristics such as socioeconomic status, race/ethnicity, and education level. These associations may sometimes influence who engages with EMA protocols and the types of contextual data captured. Despite growing interest in these patterns, no review to date has synthesized evidence on how SDoH relate to EMA compliance and engagement.

**Objective:**

We conducted a scoping review to study two research questions: (R1) how EMA compliance rates in health studies can differ across SDoH and (R2) what types of SDoH have been identified through EMA health studies.

**Methods:**

Following PRISMA-ScR guidelines, we searched PubMed, Web of Science, and EBSCOhost using two sets of queries targeting EMA and its relationship to SDoH. Eligible studies were peer reviewed, were published in English between 2013 and 2024, and used mobile-based EMA methods. Studies were included if they (1) reported on differences in EMA compliance by SDoH or (2) reported at least one SDoH observed or uncovered during an EMA study. We used the social ecological model (SEM) as a guiding framework to categorize and interpret SDoH across individual, interpersonal, community, and societal levels. A qualitative thematic synthesis was conducted to iteratively and collaboratively extract, categorize, and review determinants.

**Results:**

We analyzed 48 eligible studies, of which 35 addressed R1 by examining compliance patterns across various SDoH. Using the SEM, we identified 13 determinants categorized across 4 levels: individual (eg, daily routine, biological sex, age, socioeconomic status, language, education, and race or ethnicity), interpersonal (eg, social support), community and organizational (eg, social context, social acceptance, stigmatization, and youth culture), and policy or societal (eg, systemic and structural barriers). These studies described differences in EMA response rates, compliance, and dropout associated with these determinants, often among vulnerable populations. The remaining 13 studies addressed R2, demonstrating examples of the types of SDoH that EMA research can uncover, including family culture, social support, social contexts, stigmatization, gender norms, heroic narratives, LGBTQ+ culture, racial discrimination, and systematic and structural barriers.

**Conclusions:**

This scoping review illustrates how EMA compliance rates can differ across SDoH and highlights the potential of EMA to uncover social and cultural factors linked to health behaviors and outcomes. Our findings underscore the importance of integrating SDoH considerations into EMA study designs to capture context-specific sociocultural dynamics.

## Introduction

Understanding the relationship between human behavior and health outcomes is central to developing effective health interventions, informing evidence-based public health strategies, and identifying mechanisms that drive health and disease. Traditional research methods for studying this correspondence often rely on retrospective self-reports, surveys, interviews, and periodic assessments, leaving gaps in the recording and understanding of the nuanced fluctuation of individual experiences in real time. Ecological momentary assessment (EMA) is a method that repeatedly captures real-time behaviors and experiences within everyday, natural settings [1]. By repeatedly capturing self-reported variables such as emotions, activities, or symptoms as they occur, EMA can offer a more precise and context-sensitive understanding of how health-related issues unfold over time while also minimizing common methodological biases such as recall, recency, and availability bias [2].

Initially introduced in the early 1970s, this procedure was known as experience sampling and later more commonly referred to as EMA [3-5]. In the 4 decades since its inception, the primary vehicles of EMA data collection have been constantly evolving, moving from paper and pencil to pagers, palmtop computers, and, most recently, smartphones. Nowadays, using mobile phones for EMA data collection has become increasingly prevalent, primarily due to the widespread availability of mobile devices [6]. EMA is a popular research method applied to health-related studies to better understand phenomena such as substance use, depression, suicide, sleep, and physical activity [6-9].

The use of EMA in health-related studies is increasingly prominent due to the ubiquity of mobile phones in everyday life [10-12]. When conducting EMA studies, mobile health (mHealth) technology (eg, wearable devices, mobile phones, and mobile-based apps) can monitor real-time health-related data in real-world environments [13,14], transcending the constraints of traditional research methods (eg, survey, interview) and uncovering the dynamic interplay between human behavior and health outcomes.

A sometimes-underappreciated reality of health-focused EMA research is that social determinants of health (SDoH) may be associated with how individuals engage with EMA protocols [15,16] and the underlying health outcomes that are being studied [17-19]. An increasing body of studies is beginning to examine EMA compliance related to SDoH. For example, disregarding cultural factors in the resulting analyses has been shown to lead to failures in technology-based research among Pacific Islanders [20] and hindered user engagement on platforms like WeChat among Chinese users [21]. In another study, the authors found associations between sociocultural background (eg, historically marginalized populations) and the frequency of response to EMA text messages among substance users in the community of men who have sex with men (MSM) in San Francisco [22]. In some countries, such as China, EMA has been less feasible as a method to understand behaviors surrounding substance use disorders due to concerns about privacy [23]. These studies illustrate only a small sampling of instances where SDoH influence how individuals interact with mHealth technology, particularly in EMA research [24-28]. When applied to health studies, EMA not only facilitates data collection but also provides an opportunity to uncover underlying cultural and social dynamics associated with the studied topics [20,29,30]. For instance, one study suggested that EMA can be an effective approach for investigating suicide among Black Americans, particularly in capturing the cultural influences that shape the lived experiences of Black American men [31]. It is therefore worth exploring how EMA can be leveraged as a method to glean insights into SDoH that are often invisible, underreported, or contextually nuanced.

In this scoping review, we examine two research questions: (R1) how EMA compliance (ie, response rate) can differ across SDoH and (R2) what types of SDoH are identifiable through EMA-based health studies. Compliance in the context of EMA refers to the act of consistently following the study protocol, such as responding to prompts or completing entries as scheduled [32]. To the best of our knowledge, no prior reviews summarize the interplay between SDoH and EMA. We aim to fill this gap by identifying recent literature discussing the role of SDoH in EMA research across various health behaviors such as substance use, physical activity, sedentary behavior, dietary habits, and sexual behavior.

## Methods

### Conceptual Framework

The social ecological model (SEM) is a widely used conceptual framework for understanding the complex and multifaceted factors influencing health behavior. It posits that health and well-being are shaped by factors across 4 levels—individual, interpersonal, community and organizational, and policy and societal—including personal attributes, social relationships, institutional settings, and broader systemic or policy environments [33,34]. Intrapersonal or individual factors include demographic characteristics, attitudes, and knowledge, such as sex, age, language skills, and education [35,36]. Interpersonal factors involve interaction with others surrounding one’s social networks, such as family, friends, and groups [35]. Community and organizational factors encompass rules, policies, and practices derived from communities and organizations, such as schools, workplaces, and neighborhoods [35,37]. Policy and societal factors relate to local and national policies and the social environment, such as health policies, the legal system, and social infrastructure [38]. We used the SEM to systematically organize and interpret the various types of SDoH that we encountered in our review.

### Data Collection and Preprocessing

We conducted a scoping review following the PRISMA-ScR (Preferred Reporting Items for Systematic Reviews and Meta-Analyses Extension for Scoping Reviews) guidelines [39]. Since this study follows a scoping review methodology, no formal risk of bias or quality assessment was conducted [40]. We constructed search strings related to EMA, SDoH, feasibility, and challenges on 3 different platforms: PubMed, Web of Science, and EBSCOhost. We conducted the searches in early April 2024.

Two separate search queries were developed to address our research objectives. The following search string was used for R1: ((“ecological momentary assessment” OR “EMA” OR “mobile-based ecological momentary assessment” OR “mEMA”) AND (“feasibility” OR “challenge” OR “barrier”) AND (“social” OR “cultural” OR “culture” OR “sociocultural”)). The following search string was used for R2: ((“ecological momentary assessment” OR “EMA” OR “mobile-based ecological momentary assessment” OR “mEMA”) AND (“social” OR “cultural” OR “culture” OR “sociocultural”) AND (“digital health” OR “mobile health” OR “mHealth”)). We acknowledge that these search strings most likely do not capture every single research study involving the intersection of SDoH and EMA. Given the large volume of papers in this field, our search strings yielded a sampling of the types of papers that explore our research questions, providing valuable examples but not a comprehensive list of EMA studies involving SDoH-related factors. Full details of the search terms used for each electronic database are provided in Multimedia Appendix 1.

All database searches were conducted by the first two coauthors. We filtered articles in 3 phases and organized them using the PICO Portal, an online literature screening platform [41]. The first phase involved uploading all results to the PICO Portal and removing duplicates. The second phase involved reviewing the title, abstract, and keywords obtained from the databases searched to determine eligibility for full-text review, using predefined inclusion and exclusion criteria. These criteria were developed by the first two authors and agreed upon by all authors prior to the screening process to ensure consistency and transparency. The third phase included reviewing the full text of the potentially eligible articles by obtaining PDF documents from the 3 databases. The study selection for full-text review was performed in an iterative and collaborative process. The first author conducted the initial review, which was subsequently examined by the second author for consistency with the inclusion and exclusion criteria. Any disagreements during the screening process were resolved through discussion to reach a consensus.

After extracting relevant data from the selected studies, we used the SEM to categorize the identified sociocultural determinants. For the thematic analysis, we used an iterative and collaborative coding approach. The first author developed the initial coding framework and applied preliminary codes to the selected studies. The second author independently reviewed and refined the codes and thematic groupings to ensure consistency and comprehensiveness. While we did not use any specialized qualitative data analysis software, we maintained a structured coding matrix using the Tags and Notes functions of PICO Portal and Microsoft Excel to track emerging themes, category labels, and corresponding excerpts from the literature. Any discrepancies were discussed collaboratively among all authors until consensus was reached.

### Ethical Considerations

This study did not require ethics approval as it was a scoping review of published literature and did not involve human participants, in accordance with the policies of the University of Hawaiʻi [42].

### Terminology Mapping for EMA Compliance

Across the reviewed studies, various terms were used to describe engagement with EMA studies, including “compliance,” “compliance rate,” “response rate,” “completion rate,” “retention rate,” “adherence,” “EMA participation,” “response completion rate,” “response compliance rate,” and “noncompletion rate.” In these studies, “compliance” generally refers to the extent to which participants followed the EMA protocol by providing responses when prompted throughout the study period. The “compliance rate” represents the proportion of EMA surveys that participants answered out of the total number of valid prompts delivered, indicating whether they responded as expected. Prior studies tend to use the terms interchangeably. For example, in [43], it is stated as follows: “Compliance measures the total number of prompts answered out of those received.” In [44], it is mentioned as follows: “Compliance rate was defined as the number of answered EMA surveys divided by the number of EMA surveys prompted when the mobile phone was powered on and charged.” Similarly, the “response rate” is often used interchangeably with the “compliance rate,” reflecting the proportion of EMA survey questions answered within the required timeframe, either overall or for a specific subunit, such as daily rates. For example, in [6], it is noted as follows: “Total response rates were assessed by calculating the percentage of answered prompts per day*.*” Similarly, in [45], it is reported as follows: *“*Of the 173 new service users, 43.9% (n=56) responded to the text questionnaire.”

The “completion rate” typically indicates the proportion of participants who fully completed individual EMA surveys once started and whether they finished the entire planned study period, including any final follow-up assessments. For example, in [44], it is reported as follows: “Overall, 2862 R-EMA+CS-EMA surveys were completed (ie, all questions answered) of the 2907 survey prompts that were answered, yielding an EMA survey completion rate (once started) of 98.5%.” Other related terms include “response compliance rate” and “response completion rate.” The “noncompletion rate” indicates dropouts or extended periods without responding. For example, in [46], the researchers mentioned the following: *“*We used survival analysis methods to calculate the number of study days that transpired before a participant failed to send complete responses to EMA surveys for at least seven consecutive days (ie, time to first weeklong or more EMA survey noncompletion) .....”

The “retention rate” refers to the proportion of participants who stayed in the study and provided sufficient valid days of data to be included in the final analysis. “Adherence” reflects how well participants maintain their participation throughout the entire study duration, often quantified by metrics such as the total number of surveys completed, the pattern of daily response rates, and retention until the end of the protocol. “EMA participation” signifies that a participant initiated the EMA protocol and provided at least some responses, regardless of whether they completed the entire set of prompts. These terms were typically used interchangeably in the studies and mapped onto our definition of EMA-related “compliance” without being explicitly defined as standalone concepts.

To enable consistent synthesis and comparison, we harmonized these various terms under a unified construct of “EMA compliance,” which we define as the proportion of valid EMA prompts or surveys to which a participant provides a timely response throughout the study period, reflecting their moment-to-moment adherence to the study’s data collection schedule. We used the terms “compliance” and “compliance rate” interchangeably in MultimediaAppendices 2^14^. When original studies reported engagement using alternate terminology, we interpreted the meaning based on context (eg, whether it referred to daily response behavior, item-level completion, or sustained participation) and mapped it accordingly to our compliance definition. This mapping ensured conceptual consistency in our review despite variations in terminology used within the reviewed papers.

### Selection Criteria

#### Inclusion Criteria

Studies were eligible for this review if all of the following inclusion criteria were met: (1) studies largely focused on using EMA in a health-related context, (2) studies examined how EMA compliance rates varied across social or cultural factors, reported any social or cultural phenomena observed during the study, or discussed any emerging sociocultural practices relevant to the research context; (3) studies examined a health-related topic; (4) studies were written in English; (5) studies were published between January 2013 and April 2024; and (6) studies were published in a peer-reviewed original research article.

#### Exclusion Criteria

The exclusion criteria for this review were as follows: (1) the study was not an EMA study or not associated with health-related issues; (2) there was no meaningful discussion regarding SDoH in the EMA studies, even if related terms were mentioned; (3) EMA studies were not based on mobile devices (eg, mobile apps, text message, and phone calls) but were conducted using personal computers or other devices not considered to be mobile; (4) EMA studies reported sociocultural phenomena, yet they were unrelated to health; (5) studies were a protocol, review, proposal, or presentation summary; (6) studies were published before 2013; or (7) studies were published in languages other than English.

Given the significant advancements in mobile technology adoption and EMA research over the past decade, older studies (published before 2013) were excluded. Additionally, discussions regarding the use of smartphone capabilities to assess health or lifestyle have garnered increased attention in modern research articles [14]. Such studies reflect the modern landscape of EMA methodologies that leverage smartphone-based data collection, which aligns with the focus of this review.

## Results

### Included Studies

**Figure 1.**
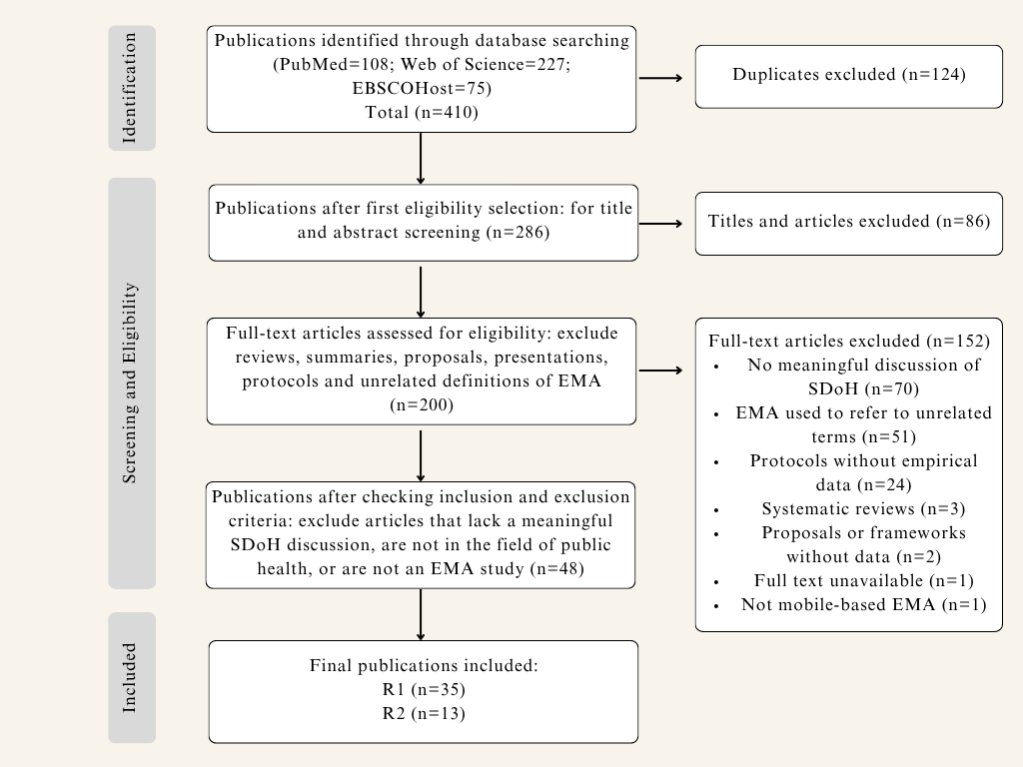
PRISMA-ScR flow diagram illustrating the study selection process of this scoping review. EMA: ecological momentary assessment; SDoH: social determinants of health.

A total of 410 papers were initially identified. After removing 124 duplicates, 286 publications were screened based on title, keywords, and abstract. Of these, 200 papers were selected for a full-text review. Following full-text assessment, 48 studies passed the inclusion and exclusion criteria and were included in the final review. Among them, 35 studies discussed 13 different SDoH, while the remaining 13 demonstrated instances where EMA research can help uncover SDoH. Figure 1 depicts the study selection process followed in this literature review, outlining the inclusion and exclusion criteria in accordance with PRISMA-ScR guidelines.

The 48 studies included in this review spanned a diverse range of populations, study settings, and health conditions. Most studies were conducted in North America, with the United States accounting for the majority (n=31) and a study from Canada (n=1) as well. There was modest representation from Europe (n=10), including studies from the UK (n=1), Sweden (n=1), Spain (n=1), Germany (n=1), France (n=1), Denmark (n=1), the Czech Republic (n=1), Austria (n=1), and the Netherlands (n=2). Asia was represented by 3 studies (n=3), with one study each from Vietnam, India, and Korea. Australia contributed 2 studies, including one comparative study between Australia and Taiwan. One study was conducted in South Africa. Across studies, sample sizes varied widely, ranging from small pilot cohorts of fewer than 20 participants (n=9) to large-scale samples of over 1000 participants (n=1). Most studies fell between 20 and 49 participants (n=20), with additional representation in the 50‐99 (n=9), 100‐199 (n=6), and 200‐499 (n=3) ranges. Study populations ranged widely and included adolescents (n=8), older adults (n=5), people who inject drugs (n=1), university students (n=3), and marginalized communities such as LGBTQ (n=4) and unhoused individuals (n=2), and individuals with chronic health conditions, including diabetes (n=2), HIV (n=2), mental health conditions (n=17), and hearing impairment (n=2). Demographic diversity was reflected across age, race, and ethnicity, including Black/African American (n=7), Hispanic/Latino (n=2), Asian (n=3), White (n=12), Pacific Islander (n=1), and mixed-race groups (n=23), as well as across gender identity, including cisgender men and women (n=41), men who have sex with men (n=4), transgender women (n=1), and nonbinary individuals (n=2).

Study designs ranged from randomized controlled trials (n=1) and quasi-experimental designs (n=3) to cross-sectional studies (n=1), longitudinal observational studies (n=1), and instrument development and validation studies (n=1). Most of the studies (n=25) used quantitative feasibility or pilot observational designs. Additionally, 9 studies used mixed-methods approaches, incorporating techniques such as focus groups (n=2), semistructured interviews (n=3), and diary-based narratives (n=1) to enrich the contextual understanding. The duration of EMA data collection ranged from 6 hours to 90 days, most commonly 7 days (n=13), 14 days (n=11), and 30 days (n=6). A few studies extended to 21 days (n=4) or 84‐90 days (n=4), while others used shorter spans, such as 10 days (n=3) or 3‐5 days (n=2). One study had a duration as short as 6 hours, and others reported variable ranges like 14‐50 days or 24‐35 days. Recruitment strategies included school recruitment (n=4), clinic recruitment (n=5), community outreach (n=5), social media (n=4), existing cohorts (n=7), and mixed approaches combining 2 or more of these methods (n=23). Across studies, eligibility criteria commonly included smartphone access, English literacy, and absence of cognitive or medical impairments that would interfere with EMA participation.

### R1: Reported Differences in EMA Compliance Rates by SDoH

#### Overview

The first research question investigates whether and how compliance rates vary across different SDoH in EMA health studies. We identified 35 of the 48 eligible articles that examined differences in EMA adherence and data completeness across various SDoH-related factors. Using the SEM as a guiding framework, we categorized these determinants into four distinct groups, following the 4-level SEM: (1) individual, (2) interpersonal, (3) community and organizational, and (4) policy or societal. These categories facilitated the identification of patterns in EMA compliance rates across diverse populations and contexts. For each selected determinant, see MultimediaAppendices 2^14^ for details on the study topic, population, key findings, and notable compliance statistics.

#### Individual-Level Determinants

##### Summary

We identified several factors mapped to the individual level based on how prior literature defines their proximity to personal agency, biological characteristics, and demographic identity. Sex, age, race, and ethnicity [37,47,48] are typically categorized as biological or demographic attributes that shape individual behavior. Meanwhile, determinants like lifestyle behaviors such as daily routines [49] represent personal choices and habits formed through individual experiences and daily routines. In addition, language, education, and socioeconomic status (SES) are often associated with an individual’s skills, capacities, and background resources, which may shape engagement in health intervention [35,37].

##### Daily Routines

Daily routines were associated with responsiveness to EMA prompts (Table S1 in Multimedia Appendix 2). Eight studies indicated preferences or dislikes regarding the timing of the EMA prompt delivery. For example, participants were more likely to respond in the mornings [44,50,51], during lunch breaks [52], evenings [53-55], weekdays [51], and weekends [50,52,56]. Four studies noted that EMA prompts could disrupt daily activities, such as during school hours [57,58], when engaged in activities like napping or driving [58], or during heavy workloads [6,59].

##### Biological Sex

Eight studies reported associations between sex and EMA compliance (Table S2 in Multimedia Appendix 3). In this review, we distinguish between sex and gender norms, with sex referring to biological characteristics and gender relating to identity and behavior [60,61]. Seven studies reported that female participants tend to have a higher compliance rate than male participants [29,45,56,59,62-64]. However, some studies found that male participants provided more responses in certain contexts. For example, one study reported that female participants are less likely to carry mobile phones due to their tight jeans [44]. This highlights that the role of biological sex in EMA participation may vary depending on contextual factors.

##### Age

Eight studies highlighted age-related differences in EMA compliance rate (Table S3 in Multimedia Appendix 4). Six studies reported that older adults maintain a higher compliance rate [29,45,51,59,65,66]. One possible explanation made by the authors is that older individuals may have more time [66], while younger participants who are employed and have variable work schedules were sometimes associated with lower compliance [65]. By contrast, one research work pointed out that older individuals may face multiple challenges, including being less familiar with technology [53], and another study reported that older people can have a hard time hearing the EMA alerts [67].

##### Socioeconomic Status

Eight studies reported that SES can be associated with EMA compliance rate in health-related EMA studies (Table S4 in Multimedia Appendix 5). Three main subfactors emerged in this category: housing, employment, and income. Three studies found that lower income negatively affected EMA compliance [46,68,69], while two studies reported that those working part-time [29] or who are unemployed [70] may have higher compliance levels than employed participants due to increased “free time.” Similarly, unemployment [52,69], a lack of stable housing [43,46,68], and living in a community with high violence [69] contributed to difficulties finding a place to charge devices or connect to the internet, further complicating compliance rates. Employment settings that restrict cell phone access can pose another barrier to compliance [65].

##### Language

Four studies emphasized the importance of language usage in EMA studies (Table S5 in Multimedia Appendix 6). Specifically, MSM participants perceived using LGBTQ-friendly language as highly important and that use of patronizing and superficial language made participants feel targeted, stereotyped, and further alienated [65]. In non–English-speaking countries, language posed a barrier when using the Fitbit App, particularly for older participants [62]. Questions framed from a negative perspective, such as “Have you thought about killing yourself since the last prompt?” can lead suicidal participants to negative mood states and reduce their compliance rates [31]. A study among Spanish-speaking psychiatric outpatients reported higher EMA compliance compared to a fully remote national sample of young adult Spanish speakers, suggesting that in-person, language-concordant support and culturally tailored procedures may enhance adherence [71].

##### Education

Four studies discussed education-level association with EMA participation (Table S6 in Multimedia Appendix 7). Among them, two studies found that higher education led to a higher compliance rate for EMA studies [72,73], whereas lower education levels correlated with lower compliance rates [46,53]. Lower levels of education can sometimes be associated with poor experience with new technologies [53].

##### Race and Ethnicity

Two studies described racial and ethnic patterns in EMA compliance rates (Table S7 in Multimedia Appendix 8). One study found that higher compliance was observed among non-White participants, specifically individuals of non-Hispanic ethnicities, including Asian, Pacific Islander, Native American, Black/African American, mixed race, and unidentified race [63]. Another study reported that histories of marginalization and deep-seated distrust of social service systems may discourage participation in EMA compliance [43].

### Interpersonal Level Determinants

#### Summary

The interpersonal level of the SEM focuses on close social relationships, such as those with family, peers, or partners, which may be associated with how individuals engage with EMA protocols. In line with prior SEM-based research [74], we categorized social support as an interpersonal determinant as it involves emotional and instrumental support through social networks that can shape participants’ compliance in EMA studies.

#### Social Support

Five studies mentioned that EMA compliance rates are associated with social support, including from family, friends, or the research team (Table S8 in Multimedia Appendix 9). Participants tend to respond to more EMA prompts when participating in the study with their family members simultaneously [50]. Moreover, receiving support from the research team, such as knowing the staff, receiving training, and having an example of feedback reports at the beginning of a study, can positively affect the compliance rate [62,67,75]. By contrast, another study noted that couples are less likely to respond to EMA prompts if they go out together [76]. This finding highlights the complexity of interpersonal dynamics in EMA participation, where supportive relationships may aid engagement in some contexts but reduce compliance rates in others due to shared distractions or privacy concerns.

### Community- and Organizational-Level Determinants

#### Summary

This level of the SEM encompasses the settings in which social relationships occur, including norms, social networks, and environmental contexts like schools, neighborhoods, and workplaces. Based on prior research [37,77], we included youth culture, social acceptance, social context, and stigmatization under this level as they reflect shared norms, cultural practices, and institutional influences that shape behavior within communities.

Youth culture and social acceptance are grounded in communal ideologies, shared values, and peer dynamics that regulate acceptable behavior [77]. Social context, including schools, churches, and neighborhoods, offers the environmental backdrop that mediates accessibility, privacy, and social risk in EMA participation [36]. Finally, stigmatization is often reinforced through institutional structures (eg, health care settings), where it may create barriers to care and limited access to services based on prevailing social norms [78].

#### Social Context

Social context refers to situational or environmental conditions that may affect the feasibility of responding to prompts, particularly when mobile phone use is seen as inappropriate or intrusive in certain contexts. Four studies reported that social context could be a barrier to EMA compliance due to concerns about disrupting others, respecting sociocultural norms, stigmatization, and privacy concerns (Table S9 in Multimedia Appendix 10). Participants reported difficulties or discomfort responding to prompts when in social settings where mobile phone use was deemed inappropriate, such as during religious ceremonies, formal gatherings [52], or environments requiring active engagement, like meetings or social events [58,79]. When researchers call participants, privacy concerns and apprehension can increase if participants are surrounded by others [66].

#### Social Acceptance

Social acceptance relates to participants’ perceptions of the topic being measured, especially in cases where the topic is sensitive or stigmatized, which may lead to fears of judgment, underreporting, or misreporting. Social acceptance of the behavior studied can affect how accurately participants report stigmatized or sensitive issues (Table S10 in Multimedia Appendix 11). For example, participants misreported behavior perceived as immoral or taboo, such as sexual activity and alcohol use [69,80,81]. Conversely, one study found that participants overreported taboo sexual behaviors since such behaviors were easier to report via electronic delivery methods [80], suggesting that electronic reporting can lower barriers to disclosure when anonymity or privacy is enhanced.

#### Stigmatization

Stigmatization is a barrier to using EMA studies for data collection (Table S11 in Multimedia Appendix 12). It involves the construction of a devalued sociocultural identity, marking certain characteristics [82]. For instance, in Vietnam, where autism is socially and culturally stigmatized, the use of EMA for self-reports is associated with introversion, which is considered a sign of autism [65]. Consequently, participants often prefer engaging in interviews when providing qualitative data rather than using their mobile phones for self-reports. Similarly, participants with mild cognitive impairment experience discomfort or even apprehension responding to EMA prompts when they are surrounded by friends, family, or public spaces [66].

#### Youth Culture

Youth culture, particularly digital communication habits, plays a significant role in shaping EMA compliance among adolescents (Table S12 in Multimedia Appendix 13). For instance, one study found that while texting is an effective and familiar mode of engagement for youth, it also introduces unique challenges [83]. EMA participation was occasionally disrupted by concurrent messaging activities, such as texting with family or friends, highlighting the multitasking nature of youth digital culture and the competition for attention within mobile environments. Additionally, to maintain participant engagement, the research team needed to become familiar with youth-specific texting shorthand (eg, “<x_x>”), emphasizing the importance of culturally and linguistically tailoring of EMA tools to align with youth communication norms.

### Policy- and Societal-Level Determinants

#### Summary

The outermost layer of the SEM includes broad systemic and structural factors shaped by national, institutional, or policy-level forces. We classified structural and systemic barriers under this level based on literature that associates them with inequities in access to healthcare, digital infrastructure, and legal systems [37,38].

#### Systemic and Structural Barriers

Social systems and structural barriers, such as incarceration, unstable housing, economic hardship, and device loss, can lead to a higher rate of noncompletion and even dropout [46,69] (Table S13 in Multimedia Appendix 14). Young MSM and transgender women (TW) living with HIV in San Francisco faced significant systemic inequities (eg, incarceration and competing medical treatment needs), preventing their participation in EMA research [46]. This underscores the need to account for these contextual challenges when designing EMA studies targeting vulnerable groups.

### R2: Types of SDoH Uncovered Through EMA Studies

Our second research question explores examples of the types of SDoH that have been identified through prior EMA studies. Of the 48 studies meeting our eligibility criteria, 13 demonstrated how EMA methods have been used to observe or uncover SDoH-related insights. Examples of the types of SDoH uncovered in the reviewed studies include (1) family culture and social support at the interpersonal level of the SEM, (2) social contexts, stigmatization, gender norms, heroic narratives, and LGBTQ+ culture at the community and organizational level of the SEM, and (3) racial discrimination and systemic and structural barriers at the policy and societal level of the SEM. Collectively, these examples illustrate the range of sociocultural dynamics documented through EMA methods.

On the interpersonal level, 3 prior EMA studies [20,84,85] have examined the relationship between family culture and health-related issues. In one study, it was identified that Chinese adolescents with autism required assistance living alone when transitioning from school as they commonly reside with parents and family, in contrast to their counterparts living in Western cultures [84]. In another study involving 42 participants (73.8% female), negative family events appeared to increase the likelihood of suicidal ideation [85]. Another study found that compared to other ethnic groups, Pacific Islander populations are less likely to smoke at home [20]. One EMA research study revealed that real-time social support from research teams can potentially decrease loneliness over time after participating in an EMA study [86]. Lastly, another study showed that, unlike perceived social support evaluated through surveys, actual social support can be assessed in real time to better understand diabetes self-management [87].

On the community and organizational level, four research studies revealed that social context, as social facilitation, could affect drinking habits [30,88,89] and cannabis use [90]. Three reviewed studies uncovered stigmatization related to health issues, including social exclusion due to schizophrenia [86], social media ostracism, and emotional impact [91], as well as stigmatization against people with autism [84]. Some studies reported how gender norms and cultural narratives intersect with health behaviors. In this study, gender norms represent culturally and socially reinforced expectations about roles and behavior that are sustained at the community level [37]. For example, female individuals with autism were more likely to engage in social activities due to cultural beliefs and gendered social expectations [84]. Another study found that teenagers recovering from brain injuries responded positively to heroic narratives, which were framed as culturally resonant models of resilience [92]. One study found that within the LGBTQ+ community, participants were more likely to share information in familiar settings such as gay bars, clubs, or private residences, where they felt a greater sense of comfort and safety [89].

On the policy and societal level, two studies found that EMA can help surface macrolevel SDoH, such as racial discrimination, system and structural barriers, and associated issues, including racial discrimination and physical activity [93], and resource barriers and geographic isolation [94]. Detailed information about each reviewed paper is provided in Table S14 in Multimedia Appendix 15.

## Discussion

### Principal Findings and Comparisons With Prior Work

We conducted a scoping review to investigate how EMA compliance rates vary across SDoH and the types of SDoH observed in health-related EMA studies from 2013 to 2024, using PubMed, Web of Science, and EBSCOhost. Guided by the conceptual framework of the SEM, we identified 35 studies that describe associations between SDoH and EMA compliance, including (1) daily routines, biological sex, age, SES, language, education, and race/ethnicity at the individual level of the SEM, (2) social support at the interpersonal level, (3) social context, social acceptance, youth culture, and stigmatization at the community and organizational levels, and (4) system and structural barriers at the policy and societal level. We also found 13 articles that demonstrated that EMA health studies can help uncover SDoH, with examples including family culture, social support, social contexts, stigmatization, gender norms, heroic narratives, LGBTQ+ culture, racial discrimination, and systemic and structural barriers. Because this is a scoping review, findings describe associations; observed differences in EMA response likely reflect design frictions and structural context rather than intrinsic participant traits.

To our knowledge, this is the first review that explores how EMA compliance rates may differ across SDoH. While SDoH are widely studied in relation to health outcomes, their relationship with EMA adherence and data completeness is less established and appears highly context-specific. Our review shows that EMA compliance can vary based on the topics studied, target population, sociocultural factors, and other study design features, potentially reflecting underlying sociodemographic and cultural differences. While not exhaustive, this review establishes a foundation for future research to further investigate the role of SDoH across multiple levels in EMA health studies.

Our findings partially align with prior research demonstrating that SDoH, such as lifestyle (daily routines), biological sex, age, SES, education, social support, social context, and systemic barriers, can influence individual behavioral patterns and health outcomes [95-97]. In contrast to previous reviews, this review, to our knowledge, is the first to explore across a broad array of studies how such factors may be associated with EMA compliance.

However, our findings also reveal inconsistencies in prior research concerning the impact of SDoH on both health outcomes and EMA compliance. For instance, while some studies reported that unemployment hinders compliance due to instability or competing demands [52,69], others suggested that unemployed individuals may exhibit higher compliance rates due to greater availability of time [70]. These types of discrepancies highlight the context-dependent nature of SDoH, suggesting that SDoH and EMA engagement are not uniform and cannot be understood in isolation from the cultural factors, target populations, and specific health issues being studied. Additionally, our analysis indicates that some determinants may serve as stronger predictors of EMA compliance than others, depending on the study context.

### Limitations

This review has several limitations: (1) We may have overlooked some articles due to the choice of search strings and search dates. It is possible that prior studies were not included due to the selection of keywords and abstract expressions, journal or indexing bias, as well as publication dates. For example, only 5 papers were retrieved if we directly used the search string ((*“*ecological momentary assessment*”*)) AND (*“*feasibility*”*) AND *(“*cultural*”* OR *“*culture*”*)). However, when we conducted hand searches for additional relevant studies, we found a few studies that discussed how SDoH can influence the feasibility and acceptability of using EMA in health. This is likely because those studies use health-oriented or method-centered keywords without explicitly mentioning sociocultural terms. This implies that SDoH are not always one of the primary objectives when conducting EMA studies, making them easy to miss. To further illustrate this limitation, we provide some examples of articles in Table S15 in Multimedia Appendix 16 that cannot be retrieved using our search string, despite addressing SDoH. (2) Most included studies were conducted in Western, high-income countries, which may limit generalizability to non-Western and low-income settings where sociocultural dynamics and technology access may differ. (3) Our interpretation of SDoH-related trends in EMA compliance may be constrained by methodological variations in the reviewed studies. For example, while certain EMA studies provide smartphones to participants to improve accessibility, others do not, potentially excluding individuals with limited digital access or literacy, which may introduce selection bias affecting low-income or older populations. (4) We acknowledge that our categorization of papers is not exhaustive, and some SDoH influencing EMA compliance may not have been examined in this scoping review.

### Future Directions

There are several important avenues of future research regarding the intersection of EMA studies and SDoH. (1) Future work should explore how multiple SDoH intersect in terms of EMA compliance patterns across populations. (2) mHealth technology has the potential to drive sociocultural transformation at both community and societal levels [98,99], much like past technological advancements that have reshaped societal norms [100,101]. For example, the rise of social media has fundamentally altered how people communicate, exchange knowledge, and share information, shaping new digital cultural norms and social interactions [102]. Therefore, public health researchers should examine how mHealth technologies and EMA practices can shape behavior and perceptions regarding health-related issues, particularly with historically marginalized groups. (3) Future EMA studies can explicitly test for differences in EMA compliance across SDoH as a primary endpoint rather than treating this question as a secondary analysis, bolstering the quantitative analysis with qualitative insights to glean reasons for the discrepancies. (4) There is a strong need for EMA studies to integrate culturally tailored approaches to improve EMA research inclusivity and relevance across diverse populations [90,103]. For example, two ongoing pilot studies are exploring the feasibility of EMA research in Native Hawaiian, Filipino, and Pacific Islander communities in Hawaii [104,105], bolstering the quantitative EMA analysis with qualitative analyses from participant interviews designed to glean culturally relevant insights. Such participatory design work, aligned with ongoing efforts in digital health equity [106-109], can and should serve as the basis for developing culturally tailored EMA studies.

### Conclusions

EMA is a popular research method in health studies, enabling researchers to monitor real-time health-related data in real-world environments that involve sociocultural values and practices. This scoping review provides several examples of studies where EMA compliance rates differ across SDoH as well as examples of EMA studies being used to uncover SDoH.

## Supplementary material

10.2196/69831 Multimedia Appendix 1 Summary of database search strategy and results for identifying studies on sociocultural factors associated with mobile-based ecological momentary assessment (EMA).

10.2196/69831 Multimedia Appendix 2 Articles that reported the impact of daily routines and the timing of notifications on ecological momentary assessment (EMA) compliance, including the possible causes of improved or worsened EMA compliance rates.

10.2196/69831 Multimedia Appendix 3 Articles that reported biological sex and its role in ecological momentary assessment (EMA) compliance, including the possible causes of improved or worsened EMA compliance rates.

10.2196/69831 Multimedia Appendix 4 Articles that reported age and its role in ecological momentary assessment (EMA) compliance, including the possible causes of improved or worsened EMA compliance rates.

10.2196/69831 Multimedia Appendix 5 Articles that reported socioeconomic status and its role in ecological momentary assessment (EMA) compliance, including the possible causes of improved or worsened EMA compliance rates.

10.2196/69831 Multimedia Appendix 6 Articles that reported language and its role in ecological momentary assessment (EMA) compliance, including the possible causes of improved or worsened EMA compliance rates.

10.2196/69831 Multimedia Appendix 7 Articles that reported education and its role in ecological momentary assessment (EMA) compliance, including the possible causes of improved or worsened EMA compliance rates.

10.2196/69831 Multimedia Appendix 8 Articles that reported race and ethnicity and their role in ecological momentary assessment (EMA) compliance, including the possible cause of improved or worsened EMA compliance rates.

10.2196/69831 Multimedia Appendix 9 Articles that reported social support and its role in ecological momentary assessment (EMA) compliance, including the possible causes of improved or worsened EMA compliance rates.

10.2196/69831 Multimedia Appendix 10 Articles that reported different social contexts and their role in ecological momentary assessment (EMA) compliance, including the possible causes of improved or worsened EMA compliance rates.

10.2196/69831 Multimedia Appendix 11 Articles that reported social acceptance and its role in ecological momentary assessment (EMA) compliance, including the possible causes of improved or worsened EMA compliance rates.

10.2196/69831 Multimedia Appendix 12 Articles that reported stigmatization and its role in ecological momentary assessment (EMA) compliance, including the possible causes of improved or worsened EMA compliance rates.

10.2196/69831 Multimedia Appendix 13 Articles that reported youth culture and its role in ecological momentary assessment (EMA) compliance, including the possible causes of improved or worsened EMA compliance rates.

10.2196/69831 Multimedia Appendix 14 Articles that reported social system and infrastructure barriers and their role in ecological momentary assessment (EMA) compliance, including the possible causes of improved or worsened EMA compliance rates.

10.2196/69831 Multimedia Appendix 15 Social determinants of health (SDoH) identified in ecological momentary assessment (EMA) studies.

10.2196/69831 Multimedia Appendix 16 A demonstration of how some ecological momentary assessment (EMA) studies discuss culture without using any keywords associated with culture.

10.2196/69831 Checklist 1 PRISMA-ScR Checklist.

## Abbreviations

AHR: adjusted hazard ratio
DP: dedicated or daily participators
EMA: ecological momentary assessment
IRR: incidence rate ratio
mHealth: mobile health
MSM: men who have sex with men
NH: non-Hispanic
OP: occasional participators
OR: odds ratio
SDoH: social determinants of health
SEM: social ecological model
SES: socioeconomic status
TW: transgender women
