# Supplementary material for: Associations Between Social Determinants of Health and Adherence in Mobile-Based Ecological Momentary Assessment: Scoping Review
Source: J Med Internet Res. 2025 Sep 23;27:e69831. doi: 10.2196/69831 (PMC12456876; doi:10.2196/69831)
Supplement: Multimedia Appendix 1 [file jmir-v27-e69831-s001.docx]

**Search queries (with quotations and boolean logic):**

**R1:** ((“ecological momentary assessment” OR “EMA” OR “mobile-based ecological momentary assessment” OR “mEMA”) AND (“feasibility” OR “challenge” OR “barrier”) AND (“social” OR “cultural” OR “culture” OR “sociocultural”))

**R2:** ((“ecological momentary assessment” OR “EMA” OR “mobile-based ecological momentary assessment” OR “mEMA”) AND (“social” OR “cultural” OR “culture” OR “sociocultural”) AND (“digital health” OR “mHealth” OR “mobile health”))

| **Database** | **Filters and Limits Applied** | **Results** |
| --- | --- | --- |
| **PubMed**  Total (n=108) | - **Date range**: 2013/01/01 to 2024/04/30  - **Text availability**: Full Free Text  - **Language**: English  - **Article types** (grouped by category):  **Clinical Trials**: Clinical Trial (all phases), Randomized Controlled Trial, Controlled Clinical Trial, Pragmatic Clinical Trial, Equivalence Trial.  **Observational and Evaluation Studies**: Observational Study, Evaluation Study, Validation Study, Comparative Study.  **Technical Report**.  **Case-Based and Descriptive**: Case Reports, Classical Article, Collected Work, Interview.  **Other Clinical Studies**: Clinical Study, Clinical Conference, Multicenter Study.  **Overall**: Captures broader or unclassified article types. | R1 (n=46)  R2 (n=62) |
| **Web of Science**  Total (n=227) | - **Date range**: 2013/01/01 to 2024/04/30  - **Text availability**: Full Text  - **Document type**: Open Access articles  - **Language**: English | R1 (n=127)  R2 (n=100) |
| **EBSCOhost**  Total (n=75) | - **Date range**: 2013/01/01 to 2024/04/30  **- Text availability**: Full Text  - **Source type**: Academic Journals, Reports  - **Language**: English  - **Peer-reviewed**: Yes | R1 (n=25)  R2 (n=50) |
