# Supplementary material for: Associations Between Social Determinants of Health and Adherence in Mobile-Based Ecological Momentary Assessment: Scoping Review
Source: J Med Internet Res. 2025 Sep 23;27:e69831. doi: 10.2196/69831 (PMC12456876; doi:10.2196/69831)
Supplement: Multimedia Appendix 2 [file jmir-v27-e69831-s002.docx]

**Table S1.** Articles that reported the impact of daily routines and the timing of notifications on EMA compliance, including the possible causes of improved or worsened EMA compliance rates.

| **Study** | **Topic** | **Population** | **Findings** | **Notable Compliance Statistics** |
| --- | --- | --- | --- | --- |
| Spook et al., 2013 [6] | Using EMA to Monitor Diet and Physical Activity | Normal-weight and overweight adolescents and young adults from education schools in the Netherlands between the ages of 16 and 21 | The authors attributed participant burden and conflicting routines such as internship days and spring break to reduced participants' compliance rate. | No quantitative statistics related to internship days or spring break provided. |
| Bell et al., 2022 [45] | Using EMA to study family eating activities | Families that include at least one adult parent and one child between the ages of 11 and 18 in Los Angeles | Compliance was significantly higher in the mornings overall and higher on the weekends for the eating-event–triggered EMAs when participants did not need to go to work or school (typically on weekend days). | OR = 0.63 (afternoon vs. morning compliance)  OR = 0.61 (evening vs. morning compliance)  OR = 1.91 (compliance when another family member had responded vs. none)  OR = 0.60 (time-triggered EMAs, afternoon vs. morning compliance)  OR = 0.53 (time-triggered EMAs, evening vs. morning compliance)  OR = 2.07 (time-triggered EMAs, compliance when another family member had responded vs. none)  OR = 2.40 (eating event-triggered EMAs, weekend vs. weekday compliance) |
| Dunton et al., 2016 [46] | Using EMA to capture physical activity | Low-to-middle–income high school adolescents in grades 9 to 12 | EMA compliance rate was lower in the mornings compared to later times in the day, which may reflect participants’ inability (due to sleep) or reluctance to answer EMA prompts in the early mornings on weekends. | 84.8% compliance rate (context-sensitive EMAs)  78.8% compliance rate (random EMAs)  8.7% increased likelihood of complying per hour as the day progresses. |
| Maher et al., 2018 [47] | Using EMA to monitor physical activity | Overweight, obese, and normal-weight older adults between the ages of 60 and 98 | Overweight and obese older adults were more likely to miss a prompt on weekends. Normal weight older adults were more likely to miss prompts in the afternoon compared to the morning. | 92% compliance rate (average)  OR = 1.72 (overweight adults; weekends vs. weekdays, missing EMA prompts)  OR = 1.53 (normal weight adults, afternoon vs. morning, missing EMA prompts) |
| Soong et al., 2015 [48] | Using EMA to study tobacco use | Individuals between the ages of 16 and 40 from local colleges, offices, and popular neighborhood places (e.g., cafes, restaurants) in urban India | Participants, particularly students, are most willing and able to use their phones and respond to a prompt during lunch or break times or on weekends. | 46.87% compliance rate (momentary EMAs)  73.02% compliance rate (end-of-day EMAs) |
| Gómez-Pérez et al., 2020 [49] | Using EMA to evaluate therapy intervention | Patients with fibromyalgia between the ages of 53 and 67 | Evening compliance rate was high compared to the morning, which might be due to poor sleep quality in this population. | 45.2%-88.10% compliance rate (patient-level)  61.22% compliance rate (morning, group-level)  68.37% compliance rate (evening, group level) |
| Narziev et al., 2020 [50] | Using EMA to detect short-term depression | Individuals from  depression groups based on the Patient Health Questionnaire-9 (ages are not reported) | The response rate at 7 a.m. was quite lower than at other hours, possibly due to late wake-up times or rushing to work or school. | 38% compliance rate  (7 a.m.)  60% compliance rate  (10 a.m.)  64% compliance rate  (1 p.m.)  61% compliance rate  (4 p.m.)  60% compliance rate  (7 p.m.)  58% compliance rate  (10 p.m.) |
| Swendeman et al., 2020 [51] | Using EMA for self-monitoring of family functioning | Young adolescents between the ages of 10 and 14 and their parents | Based on the descriptive observation following the quantitative analysis of response timing, children tended to miss the morning and noontime EMAs, whereas parents tended to miss the late afternoon/early evening EMAs. Completing surveys on weekends can be difficult, as activities and schedules are less routine.  Qualitative analysis from participants also reported that end-of-day surveys were burdensome due to fatigue | No quantitative statistics related to time-of-day provided. |
| El Dahr et al., 2023 [52] | Using EMA to understand the daily experiences of parents and children | Parents and their children between the ages of 9 and 13 | Completion rates were higher on weekdays than on weekends. Between mothers and children, children’s compliance was lower on weekends than on weekdays. | 82.4% compliance rate (EMAs, weekdays)  77.8% compliance rate (EMAs, weekends, p = .02)  43.1-minute mean response time (evenings) vs  51.0-minute mean response time (mornings, p = .03) |
| Ferguson et al., 2023 [53] | Using EMA to study sugary drink consumption | African American or Black adolescents between the ages of 12 and 17 residing in Washington, DC | As per authors’ interpretation of contextual limitations not being able to use mobile phones at school limited the time windows that EMA prompts could be administered and/or completed. | 68% compliance rate (random, researcher-initiated EMAs completed at home) |
| Xu et al., 2020 [54] | Using EMA for audiology Research | Adults aged 22 to 78 years with hearing impairment (HI group) and 19 to 37 years with normal hearing (NH group) | Participants would not complete surveys when they were involved in activities, such as when they were working, in a class or meeting, driving, napping, bathing, or exercising. | Over 60% of participants reported that repetitive surveys disrupted daily activities, including during work, class, driving, and social events. |
| Kronkvist et al., 2020 [55] | Using EMA for daily assessment of crime fear | College students at Malmö University | The average participant only answered surveys for a handful of days before dropping out, which may have been due to a perceived heavy workload. | 28%–31% compliance rate (signal-contingent EMAs, full sample)  21%–24% compliance rate (signal-contingent EMAs, Occasional Participators or OP group)  49%–53% compliance rate (signal-contingent EMAs, Dedicated Participators or DP group)  61%–68% compliance rate (daily assessment, full sample)  53%–59% compliance rate (daily assessment, OP group)  77%–85% compliance rate (daily assessment, DP group) |
