# Supplementary material for: Associations Between Social Determinants of Health and Adherence in Mobile-Based Ecological Momentary Assessment: Scoping Review
Source: J Med Internet Res. 2025 Sep 23;27:e69831. doi: 10.2196/69831 (PMC12456876; doi:10.2196/69831)
Supplement: Multimedia Appendix 3 [file jmir-v27-e69831-s003.docx]

**Table S2.** Articles that reported biological sex and its role in EMA compliance, including the possible causes of improved or worsened EMA compliance rates.

| **Study** | **Topic** | **Population** | **Findings** | **Notable Compliance Statistics** |
| --- | --- | --- | --- | --- |
| Nam et al., 2020 [29] | Using EMA to understand biobehavioral responses to stress and racial discrimination | Middle-aged African Americans between the ages of 30 to 55 | Females showed higher EMA compliance rates. | 82.8% compliance rate (average)  87.5% compliance rate (female)  70.3% compliance rate (male) |
| Dunton et al., 2016 [46] | Using EMA to capture physical activity | Low-to-middle–income high school adolescents in grades 9 to 12 | Girls were less likely to carry mobile phones because they did not fit in their pant pockets. | Compliance rate by gender: No significant difference (p > .05).  OR = 3.11  (girls vs. boys, more likely to carry phone within reach)  OR = 0.15  (girls vs. boys, less likely to carry phone in pocket) |
| El Dahr etal., 2023 [52] | Using EMA to understand the daily experiences of parents and children | Parents and their children between the ages of 9 and 13 | Compliance was higher with girls than in dyads with boys, but no explanation was provided. | Average compliance based on number of EMA assessments completed: 24.9 (girls, SD = 6.2)  21.7 (boys, SD = 8.4, p = .02) |
| Kronkvist et al., 2020 [55] | Using EMA for daily assessment of crime fear | College students at Malmö University | More women participated in the study due to a sense of responsibility for the study topic. | OR = 2.75  (female vs. male, OP vs. abstainer, signal-contingent EMAs, p < .05)  OR = 6.50  (female vs. male, DP vs. abstainer, signal-contingent EMAs, p < .01)  OR = 6.90  (female vs. male, DP vs. abstainer, daily assessment EMAs, p < .05) |
| Elavsky et al., 2021 [58] | Using EMA to monitor physical activity | Czech adults between the ages of 50 and 74 | Internal data indicated women had a slightly higher compliance rate. | No quantitative statistics related to sex-differences provided. |
| Mackesy-Amiti et al., 2018 [59] | Using EMA to study mood and risk behavior | Young people who inject drugs (PWID) between the ages of 18 and 35 | Women were more likely to have a higher compliance than men. | 85.5% compliance rate (female)  72.2% compliance rate (male)  adjusted odds ratio (aOR) = 4.06 (female vs. male, consistent EMA compliance)  Adjusted Incidence Rate Ratio (aIRR) for number of surveys completed: 1.51  (female vs. male)  aIRR = 1.33  (female vs. male, number of days with at least one EMA response) |
| Valentiner et al., 2019 [61] | Using EMA for interval walking training | Patients between the ages of 18 and 80 with type 2 diabetes | Men had a lower compliance rate than women. | 83% compliance rate (retention-based)  90% compliance rate (female)  68% compliance rate (male) |
| Willoughby et al., 2018 [62] | Using EMA to provide adolescent sexual health service | Students between the ages of 13 and 17 in middle and high schools | Female students were more likely than male students to comply with the SMS questionnaire. | 76.1% compliance rate (female, SMS questionnaire) |
