# Supplementary material for: Associations Between Social Determinants of Health and Adherence in Mobile-Based Ecological Momentary Assessment: Scoping Review
Source: J Med Internet Res. 2025 Sep 23;27:e69831. doi: 10.2196/69831 (PMC12456876; doi:10.2196/69831)
Supplement: Multimedia Appendix 4 [file jmir-v27-e69831-s004.docx]

**Table S3.** Articles that reported age and its role in EMA compliance, including the possible causes of improved or worsened EMA compliance rates.

| **Study** | **Topic** | **Population** | **Findings** | **Notable Compliance Statistics** |
| --- | --- | --- | --- | --- |
| Nam et al., 2020 [29] | Using EMA to understand biobehavioral responses to stress and racial discrimination | Middle-aged African Americans between the ages of 30 to 55 | Older participants had higher compliance rates. | Higher compliance (response rate) significantly associated with older age (p = .03) |
| Maher et al., 2018 [47] | Using EMA to monitor physical activity of older People | Overweight, obese, and normal-weight older adults between the ages of 60 and 98 | Based on the analysis of logged response data, done by authors, older adults demonstrated high compliance rate (~92%), though some had difficulty hearing the EMA alerts. | No quantitative statistics related to age disparities provided. |
| Gómez-Pérez et al., 2020 [49] | Using EMA to evaluate therapy intervention | Patients with fibromyalgia between the ages of 53 and 67 | Older people were less familiar with technology. | 88% compliance rate (younger participants, ages 50–64)  45%–69% compliance rate (older participants, ages 65+) |
| Kronkvist et al., 2020 [55] | Using EMA for daily assessment of crime fear | College students at Malmö University | The compliance rate was higher among senior students. | Older participants were more likely to belong to the high-compliance Dedicated Participant (DP) group.  OR = 1.26 (each additional year of age, signal-contingent surveys, consistent EMA compliance, p < .01)  OR = 1.16 (each additional year of age, daily assessments, consistent EMA compliance, p < .05) |
| Willoughby et al., 2018 [62] | Using EMA to provide adolescent sexual health service | Students between the ages of 13 and 17 in middle and high schools | High school seniors had a higher compliance rate compared to younger students. | Age comparison across survey formats:  Mean age of participants complying SMS survey: 15.14 years  No quantitative statistics related to age disparities provided. |
| Trang et al., 2022 [63] | Using EMA to monitor the relationship between mental distress and HIV risk | MSM between the ages of 18 and 24 in Hanoi, Vietnam | Based on qualitative analyses, the authors found that younger participants had unique challenges compared to older participants (e.g., variable work schedules and limited phone access) as barriers to responding to EMA prompts | No quantitative statistics related to age disparities provided. |
| Mattos et al., 2019 [64] | Using EMA for mood assessment | Individuals between the ages of 69 and 81 with mild cognitive impairment (MCI) | Authors speculated that older adults responded more to EMA due to having greater available time and fewer daily obligations. | No quantitative statistics related to age disparities provided. |
| Burke et al., 2022 [65] | Feasibility of app-based noncontact EMA | Experienced and technology-naïve older participants between the ages of 45 and 78 | Older users had difficulty hearing the EMA alerts. | 83.9% compliance rate (experienced participants, EMA completion)  64.1% compliance rate (experienced participants, EMA response)  53.8% compliance rate (naïve participants, EMA completion)  54.3% compliance rate (naïve participants, EMA response)  Older participants more frequently reported technical barriers to compliance (e.g., not receiving survey alerts) (p= .008) |
