# Supplementary material for: Associations Between Social Determinants of Health and Adherence in Mobile-Based Ecological Momentary Assessment: Scoping Review
Source: J Med Internet Res. 2025 Sep 23;27:e69831. doi: 10.2196/69831 (PMC12456876; doi:10.2196/69831)
Supplement: Multimedia Appendix 5 [file jmir-v27-e69831-s005.docx]

**Table S4.** Articles that reported socioeconomic status and its role in EMA compliance, including the possible causes of improved or worsened EMA compliance rates.

| **Study** | **Topic** | **Population** | **Findings** | **Notable Compliance Statistics** |
| --- | --- | --- | --- | --- |
| Nam et al., 2020 [29] | Using EMA to understand biobehavioral responses to stress and racial discrimination | Middle-aged African Americans between the ages of 30 to 55 | Participants who worked part-time had higher compliance rates than their counterparts. | Part-time employment was associated with significantly higher overall compliance (p=.02). |
| Soong et al., 2015 [48] | Using EMA to understand tobacco behavior in urban India | Participants between the ages of 16 and 40 | Unemployed groups (e.g., students) were less likely to comply as they might be in less appropriate areas for mobile phone use. | Being employed predicted higher momentary (p=.01) and end-of-day (p=.48) compliance rate. |
| Trang et al., 2022 [63] | Monitoring the relationship between mental distress and HIV risk | MSM between the ages of 18 and 24 in Hanoi, Vietnam | Based on qualitative observation, the authors found that employed participants may have a lower compliance rate because of irregular or demanding work schedules as they did not have phone access at work. | No quantitative statistics related to employment differences provided. |
| Biello et al., 2020 [66] | Real-time co-occurrence of drug use and harm reduction service | People between the ages of 30 and 47 who inject drugs | Authors interpreted that housing instability and disruptions in cell phone access can be key socioeconomic barriers to EMA compliance. | Higher compliance for participants with higher education (p=.005)  No quantitative statistics related to housing status or cell phone accessibility provided. |
| Turner et al., 2019 [67] | Feasibility of using EMA for data collection | Young MSM and TW between the ages of 18 to 34 living with HIV in San Francisco | Participants who lived in temporary/transitional housing with lower income had a lower compliance rate. | aHR = 1.78 (participants in temporary/transitional housing vs. stable housing, long-term EMAs compliance, p = .03) |
| Yang et al., 2015 [68] | Using EMA to study alcohol use | African American MSM between ages of 27 and 62 in Baltimore | One participant missed EMA due to incarceration. Authors interpreted unemployment, low-income status, incarceration, and community violence as potential barriers to EMA compliance. | No quantitative statistics related to unemployment, income, incarceration, and community violence provided. |
| Song et al., 2023 [69] | Leisure activity participation | Autistic adults of 18 years or older | Autistic adults with a paid job had higher compliance rates. | Average compliance:  90.2%  94.8% compliance rate (paid full-time job, EMAs)  83.9% compliance rate (paid part-time job, EMA, p = .01) |
| Semborski et al., 2022 [70] | Feasibility of using EMA with homeless | Young adults without stable housing | Unhoused participants had difficulty in charging the devices, bringing increased stress and concern of being tracked. | Unhoused participants reported practical barriers to compliance, including phone charging difficulty (p= .02), stress/anxiety (p=.02), and daily interference (p< .001) |
