# Supplementary material for: Associations Between Social Determinants of Health and Adherence in Mobile-Based Ecological Momentary Assessment: Scoping Review
Source: J Med Internet Res. 2025 Sep 23;27:e69831. doi: 10.2196/69831 (PMC12456876; doi:10.2196/69831)
Supplement: Multimedia Appendix 6 [file jmir-v27-e69831-s006.docx]

**Table S5**. Articles that reported language and its role in EMA compliance, including the possible causes of improved or worsened EMA compliance rates.

| **Study** | **Topic** | **Study Population** | **Findings** | **Notable Compliance Statistics** |
| --- | --- | --- | --- | --- |
| Adams et al., 2024 [31] | Using EMA to assess suicide | Black men between the ages of 18 and 34 with suicidal thoughts and behaviors | Qualitative analysis showed compliance reduced by deficit-framed suicide questions, which negatively affected mood and engagement. Participants recommended more positively framed and flexible EMA content. | No quantitative statistics related to language differences provided. |
| Elavsky et al., 2021 [58] | Using EMA for real-time behavior monitoring | Czech adults between the ages of 50 and 74 | Authors noted that participants in non-English-speaking regions faced occasional challenges with app usability and Fitbit settings due to language/localization gaps | No quantitative statistics related to language differences provided. |
| Silva et al., 2022 [60] | Using EMA to assess suicide risk | Spanish-speaking psychiatry adult  outpatients between the  ages of 24 and 63 years | High acceptability and engagement with Spanish-language EMA; language accessibility supported feasibility in a high-risk group | 74.05% compliance rate (average, SD = 17.61%) |
| Trang et al., 2022 [63] | Monitoring the relationship between mental distress and HIV risk | MSM between the ages of 18 and 24 in Hanoi, Vietnam | Qualitative analyses showed that participants perceived having an MSM-friendly and culturally appropriated language as highly important. | No quantitative statistics related to language differences provided. |
