# Supplementary material for: Associations Between Social Determinants of Health and Adherence in Mobile-Based Ecological Momentary Assessment: Scoping Review
Source: J Med Internet Res. 2025 Sep 23;27:e69831. doi: 10.2196/69831 (PMC12456876; doi:10.2196/69831)
Supplement: Multimedia Appendix 7 [file jmir-v27-e69831-s007.docx]

**Table S6**. Articles that reported education and its role in EMA compliance, including the possible causes of improved or worsened EMA compliance rates.

| **Study** | **Topic** | **Population** | **Findings** | **Notable Compliance Statistics** |
| --- | --- | --- | --- | --- |
| Gómez-Pérez et al., 2020 [49] | Using EMA to evaluate therapy intervention | Patients with Fibromyalgia between the ages of 53 and 67 | Authors speculated that patients with relatively low education (primary or secondary) and limited familiarity with smartphone technology might have influenced the lower-than-expected compliance. | No quantitative statistics related to education level differences provided. |
| Turner et al., 2019 [67] | Investigating the social inequity and structural barriers to complete EMA | Young MSM and TW between the ages of 18 and 34 living with HIV in San Francisco | Participants with less than a college education have a higher noncompliance rate. | aHR = 1.83 (participants with only high school education vs. some college or more),    Extended EMAs noncompletion, 95% CI: 1.16–2.89, p = .01) |
| Kirk et al., 2013 [72] | Using EMA for data collection with illicit drug users | Individuals between ages of 40 to 55 who self-reported illicit drug use and craving | People with higher education have a higher compliance rate to random prompts. | ≥80% compliance rate (participants with at least high school education, EMAs, OR = 2.07, p = .012) |
| Klaus et al., 2022 [73] | Understanding withdrawal and adherence to self-report EMA survey | Community-dwelling older aged adults between the ages of 67 and 87 years | People with higher education have a higher compliance rate. | Participants with more years of education demonstrated significantly better adherence to EMA surveys (p= .013) |
