# Supplementary material for: Associations Between Social Determinants of Health and Adherence in Mobile-Based Ecological Momentary Assessment: Scoping Review
Source: J Med Internet Res. 2025 Sep 23;27:e69831. doi: 10.2196/69831 (PMC12456876; doi:10.2196/69831)
Supplement: Multimedia Appendix 8 [file jmir-v27-e69831-s008.docx]

**Table S7.** Articles that reported race and ethnicity and their role in EMA compliance, including the possible cause of improved or worsened EMA compliance rates.

| **Study** | **Topic** | **Population** | **Findings** | **Notable Compliance Statistics** |
| --- | --- | --- | --- | --- |
| Mackesy-Amiti et al., 2018 [59] | Using EMA to study mood and risk behavior | Young people who inject drugs (PWID) between the ages of 18 and 35 | Among non-white participants, individuals of non-Hispanic ethnicities, including Asian, Pacific Islander, Native American, Black/African American, mixed race, and unidentified race, were more responsive. | 84% compliance rate (Hispanic participants, daily EMAs) vs. 94% compliance rate (other racial/ethnic groups, p = .057)  aOR = 2.43 (non-Hispanic “Other” race/ethnicity, days responded, p = .030) |
| Semborski et al., 2022 [70] | Feasibility of using EMA | Young adults without stable housing | Authors interpreted that histories of marginalization and systemic distrust, including social service systems, could discourage participation (e.g., the concern of confidentiality). | No quantitative statistics related to racial differences in EMA compliance provided. |
