# Supplementary material for: Associations Between Social Determinants of Health and Adherence in Mobile-Based Ecological Momentary Assessment: Scoping Review
Source: J Med Internet Res. 2025 Sep 23;27:e69831. doi: 10.2196/69831 (PMC12456876; doi:10.2196/69831)
Supplement: Multimedia Appendix 9 [file jmir-v27-e69831-s009.docx]

**Table S8.** Articles that reported social support and its role in EMA compliance, including the possible causes of improved or worsened EMA compliance rates.

| **Study** | **Topic** | **Population** | **Findings** | **Notable Compliance Statistics** |
| --- | --- | --- | --- | --- |
| Bell et al., 2022 [45] | Using EMA to study family eating activities | Families that include at least one adult parent and one child between the ages of 11 and 18 years in Los Angeles | Participants were more likely to answer an EMA if another family member had answered it in a similar time frame. | 89.4% compliance rate (average, family level)  89.6% compliance rate (average, individual level)  OR = 1.91 (participant response if another family member had responded recently, all EMAs)  OR = 2.07 (participant response if another family member had responded recently, time-triggered EMAs) |
| Elavsky et al., 2021 [58] | Using EMA for real-time behavior monitoring | Czech adults between the ages of 50 and 74 | Participants reported they would have been more compliant and attentive if they knew what type of feedback was possible. Authors suggested that previewing feedback reports and offering device setup/training support may improve compliance. | No quantitative statistics related to participation feedback or training provided. |
| Burke et al., 2022 [65] | Feasibility of app-based noncontact EMA | Experienced and technology-naïve older participants between the ages of 45 and 78 | Based on descriptive observations, authors infer that participants with prior EMA experience and a history of lab visits may have had stronger relationships with researchers, potentially contributing to higher participation and compliance. | No quantitative statistics related to researchers’ support provided. |
| Fortuna et al., 2022 [76] | Using EMA for peer support interventions | Patients of age 18 years of older with serious mental illness | Participants with higher levels of social support at follow-up have higher EMA compliance. | Participants completing >20% of EMA responses showed trend-level higher social support at 3 months (mean = 79.22) than those with lower compliance (mean= 59.60, p = 0.095) |
| Derrick et al., 2018 [77] | Using EMA to study smoking concessions | Single-smoker couples between ages of 18 and 55 | Couples are less likely to reply to a prompt when together in public. | 86% compliance rate (“quitters”, EMAs, when partner compliant)  30% compliance rate (“quitters”, EMAs, when partner not compliant)  OR = 1.18 (lapse report from “quitters” expecting more support vs. less or no partner support)  OR = 3.25 (lapse report from partners with higher planned support vs. lower or no planned support, own compliance) |
