# Supplementary material for: Associations Between Social Determinants of Health and Adherence in Mobile-Based Ecological Momentary Assessment: Scoping Review
Source: J Med Internet Res. 2025 Sep 23;27:e69831. doi: 10.2196/69831 (PMC12456876; doi:10.2196/69831)
Supplement: Multimedia Appendix 10 [file jmir-v27-e69831-s010.docx]

**Table S9.**  Articles that reported different social contexts and their role in EMA compliance, including the possible causes of improved or worsened EMA compliance rates.

| **Study** | **Topic** | **Population** | **Findings** | **Notable Compliance Statistics** |
| --- | --- | --- | --- | --- |
| Soong et al., 2015 [48] | Using EMA to study tobacco use | Individuals between the ages of 16 and 40 from local colleges, offices, and popular neighborhood places (e.g., cafes, restaurants) in urban India | Students were less likely to comply, possibly due to being in environments (e.g., classrooms) where phone use was inappropriate. | 46.87% compliance rate (momentary surveys)  73.02% compliance rate (end-of-day surveys)  β = –0.17 (exposure to others using tobacco, predictor of lower momentary EMAs compliance, p = .02)  β = –0.15 (exposure to anti-tobacco messages, predictor of lower momentary EMAs compliance, p = .01) |
| Xu et al., 2020 [54] | Using EMA for audiology research | Adults between the ages of 22 and 78 years with hearing impairment (HI) and 19 and 37 years with normal hearing (NH) | Qualitative analysis indicated that participants reported EMA prompts as disruptive during daily activities, especially work, driving, classes, or social events, and often preferred to snooze or skip them in these situations. | About 60% of participants reported that repetitive surveys somewhat interrupted their activities—particularly during work, driving, or social events. |
| Mattos et al., 2019 [64] | Using EMA for mood assessment | Individuals between the ages of 69 and 81 with mild cognitive impairment | Authors speculated that participants may feel discomfort in responding to EMA prompts in public or social settings (such as when surrounded by friends and families or in public places). | No quantitative statistics comparing compliance rates between participants when alone versus accompanied by others provided. |
| Schinkel-Bielefeld et al., 2020 [80] | Using EMA to evaluate hearing aids in everyday life | Individuals between the ages of 24 to 79 with hearing impairment | Participants purposefully did not bring the study phone to social situations or skipped questionnaires because doing so might be considered as inappropriate (e.g., in church, engaging in a conversation) or safety considerations outweigh the desire to respond during certain activities (such as driving). | 46% (social inappropriateness, barrier to EMAs compliance)  35% (safety reasons such as driving a car, barrier to EMAs compliance)  36% (missed triggers, barrier to EMAs compliance) |
