# Supplementary material for: Associations Between Social Determinants of Health and Adherence in Mobile-Based Ecological Momentary Assessment: Scoping Review
Source: J Med Internet Res. 2025 Sep 23;27:e69831. doi: 10.2196/69831 (PMC12456876; doi:10.2196/69831)
Supplement: Multimedia Appendix 11 [file jmir-v27-e69831-s011.docx]

**Table S10.** Articles that reported social acceptance and its role in EMA compliance, including the possible causes of improved or worsened EMA compliance rates.

| **Study** | **Topic** | **Population** | **Findings** | **Notable Compliance Statistics** |
| --- | --- | --- | --- | --- |
| Yang et al., 2015 [68] | Using EMA to study alcohol use | African American MSM between ages of 27 and 62 in Baltimore | Many people reported that answering EMA questions about drinking made them want to drink less. | 20%–31% reported that answering EMA questions about drinking made them want to drink less. |
| Dietrich et al., 2020 [81] | Using EMA to assess sexual risk | Women between the ages of 18 and 25 years old who self-reported sexual risk behavior or were at risk of HIV infection | Participants stated that they might have overreported sexual activity because of social desirability bias. Some participants hid or lost study phones to avoid stigma or theft in drug-affected households. | 6 out of 59 participants (10%) described over reporting sexual activity because of social desirability bias. |
| Willis et al., 2021 [82] | Using EMA to study sexual consent | Sexually active individuals between the ages of 18 and 39 | Authors speculated that participants were inclined to misreport certain types of behaviors (e.g., sex, alcohol use) due to the social desirability bias. | No quantitative statistics related to social acceptance and EMA compliance rate provided. |
