# Supplementary material for: Associations Between Social Determinants of Health and Adherence in Mobile-Based Ecological Momentary Assessment: Scoping Review
Source: J Med Internet Res. 2025 Sep 23;27:e69831. doi: 10.2196/69831 (PMC12456876; doi:10.2196/69831)
Supplement: Multimedia Appendix 12 [file jmir-v27-e69831-s012.docx]

**Table S11.** Articles that reported stigmatization and its role in EMA compliance, including the possible causes of improved or worsened EMA compliance rates.

| **Study** | **Topic** | **Population** | **Findings** | **Notable Compliance Statistics** |
| --- | --- | --- | --- | --- |
| Trang et al., 2022 [63] | Monitoring the relationship between mental distress and HIV risk | MSM between the ages of 18 and 24 in Hanoi, Vietnam | Qualitative analysis reported that autism stigma makes EMA self-reports less preferred than interviews. Participants valued MSM-friendly language; impersonal app design and stigma concerns (e.g., privacy, fear of judgment) lowered engagement. | No quantitative statistics related to stigma provided. |
| Mattos et al., 2019 [64] | Using EMA for mood assessment | Individuals between the ages of 69 and 81 with mild cognitive impairment | Authors speculated that receiving prompts can bring privacy-related apprehension about using EMA due to privacy concerns and fear of judgment. | No quantitative statistics comparing compliance rates between participants when alone versus accompanied by others provided. |
