# Supplementary material for: Associations Between Social Determinants of Health and Adherence in Mobile-Based Ecological Momentary Assessment: Scoping Review
Source: J Med Internet Res. 2025 Sep 23;27:e69831. doi: 10.2196/69831 (PMC12456876; doi:10.2196/69831)
Supplement: Multimedia Appendix 13 [file jmir-v27-e69831-s013.docx]

**Table S12.**  Articles that reported youth culture and its role in EMA compliance, including the possible causes of improved or worsened EMA compliance rates.

| **Study** | **Topic** | **Population** | **Findings** | **Notable Compliance Statistics** |
| --- | --- | --- | --- | --- |
| Garcia et al., 2014 [83] | Using EMA to collect real-time data that influence physical, mental, emotional, and social well-being | Latina high school adolescents between ages of 14 and 17 | Researchers observed that EMA responses via text had to be interpreted within the context of youth texting culture, e.g., <x_x> (“smiling”). EMAs competed with other messaging activities, such as texting families and friends. | No quantitative statistics related to text differences or youth culture provided. |
