# Supplementary material for: Associations Between Social Determinants of Health and Adherence in Mobile-Based Ecological Momentary Assessment: Scoping Review
Source: J Med Internet Res. 2025 Sep 23;27:e69831. doi: 10.2196/69831 (PMC12456876; doi:10.2196/69831)
Supplement: Multimedia Appendix 14 [file jmir-v27-e69831-s014.docx]

**Table S13.** Articles that reported social system and infrastructure barriers and their role in EMA compliance, including the possible causes of improved or worsened EMA compliance rates.

| **Study** | **Topic** | **Population** | **Findings** | **Notable Compliance Statistics** |
| --- | --- | --- | --- | --- |
| Turner et al., 2019 [67] | Feasibility of using EMA for data collection | Young MSM and TW between the ages of 18 and 34 years living with HIV in San Francisco | TW and MSM living with HIV faced significant structural barriers (e.g., incarceration, competing needs) that prevent them from participating in EMA research. | aHR = 1.78 (temporary or transitional housing vs. renting/owning a house, extended EMAs noncompliance, p = .03)  aHR = 1.71 (sacrificing basic needs for HIV medication, increased EMAs noncompliance, p = .02) |
| Yang et al., 2015 [68] | Using EMA to study alcohol use | African American MSM between ages of 27 and 62 in Baltimore | As per authors’ speculation, social challenges such as unemployment, low-income status, incarceration, community violence and infrastructure barrier related to device loss or theft may interfere with study participation | No quantitative statistics related to social challenges provided. |
