# Supplementary material for: Associations Between Social Determinants of Health and Adherence in Mobile-Based Ecological Momentary Assessment: Scoping Review
Source: J Med Internet Res. 2025 Sep 23;27:e69831. doi: 10.2196/69831 (PMC12456876; doi:10.2196/69831)
Supplement: Multimedia Appendix 15 [file jmir-v27-e69831-s015.docx]

**Table S14**: SDoH identified in EMA studies.

|  | **Social Determinant of Health** | | | **Topic** |
| --- | --- | --- | --- | --- |
| **Studies** | **Interpersonal Level** | **Community and Organizational Level** | **Policy and Societal Level** |  |
| Pike et al., 2016 [20] |  | Pacific islander culture; family culture |  | Using EMA to measure cigarette use |
| Fischer et al., 2023 [30] |  | Social context |  | Using EMA to understand binge drinking, its context, and alcohol use |
| Chen et al., 2017 [84] |  | Family and community culture,  gender norms, stigmatization |  | Using EMA to explore social participation |
| Husky et al., 2017 [85] |  | Family and community culture |  | Using EMA to study predictors of daily life suicidal ideation (SI) |
| Hanssen et al., 2020 [86] | Social support | Stigmatization |  | Using experience sampling to improve daily social functioning and symptoms in schizophrenia spectrum disorders |
| Wooldridge et al., 2022 [87] | Social support |  |  | Using EMA to assess psychosocial factors and self-management behaviours |
| Pennay et al., 2023 [88] |  | Social context |  | Using EMA to understand alcohol consumption while watching football |
| Wray et al., 2019 [89] |  | LGBTQ+ culture |  | Using EMA to study alcohol-drinking locations |
| Phillips et al., 2018 [90] |  | Social context |  | Using EMA to assess whether social context influences cannabis use |
| Stieger et al., 2020 [91] |  | Social context, stigmatization |  | Using EMA to study social media ostracism and emotion |
| Worthen-Chaudhari et al., 2017 [92] |  | Heroic Narratives as cultural constructs |  | Using the mHealth application for unresolved concussion symptoms |
| Nam et al., 2021 [93] |  |  | Racial discrimination | Using EMA to understand racial discrimination and physical activities (PA) |
| Browning et al., 2022 [94] |  |  | Racial discrimination, systemic and structural barriers | Using EMA to study isolation and mobility |
