# Supplementary material for: Associations Between Social Determinants of Health and Adherence in Mobile-Based Ecological Momentary Assessment: Scoping Review
Source: J Med Internet Res. 2025 Sep 23;27:e69831. doi: 10.2196/69831 (PMC12456876; doi:10.2196/69831)
Supplement: Multimedia Appendix 16 [file jmir-v27-e69831-s016.docx]

**Table S15.** A demonstration of how some EMA studies discuss culture without using any keywords associated with culture.

| **Study** | **Topic** | **Population** | **Lessons Learned** | **Keywords** |
| --- | --- | --- | --- | --- |
| Han et al., 2018 [23] | Feasibility of using EMA for substance use disorders | People between the ages of 18 and 65 who are dependent on heroin or amphetamine-type stimulant (ATS) in Shanghai | Acceptability was low, as drug addiction is treated as immoral in China. | mHealth; substance use; heroin dependence; amphetamine-type stimulant (ATS) dependence; mobile app; China |
| Hubach et al.,2021 [25] | Feasibility of using EMA for data collection | MSM between the ages of 18 and 36 who live in rural communities in Oklahoma | MSM were afraid of unwanted disclosure of their homosexuality due to a lack of identified social resources, nondiscrimination policies, and inclusive faith in rural Oklahoma. | mobile research; men who have sex with men; rural; data privacy; sexual orientation; EMA |
| Tonkin et al., 2023 [26] | Evaluating the EMA time course and exploring the predictors of compliance rates | Cigarette-using adults between ages of 43 and 63 | African American participants exhibited lower compliance rates. which may be due to daily stress and mistrust attributed to institutional racism. | ecological momentary assessment; compliance; health behavior; methodology; longitudinal; health behavior; smoking; smoker; cessation; quit; adherence; dropout; RCT; cigar; retention |
| Berge et al., 2018 [103] | Using EMA to understand parent feeding practices | Children between the ages 5 and 7 and their parents from six racial groups | EMA messages should be culturally tailored to different racial/ethnic groups, since food is culturally specific. | parent feeding practices; ecological momentary assessment; minority; low-income; immigrants |
